# Prognostic Value of Galectin-9 Relates to Programmed Death-Ligand 1 in Patients With Multiple Myeloma

**DOI:** 10.3389/fonc.2021.669817

**Published:** 2021-06-14

**Authors:** Byung-Hyun Lee, Yong Park, JI-Hea Kim, Ka-Won Kang, Seung-Jin Lee, Seok Jin Kim, Byung Soo Kim

**Affiliations:** ^1^ Department of Internal Medicine, Korea University College of Medicine, Anam Hospital, Seoul, South Korea; ^2^ Department of Biomedical Science, Graduate School of Medicine, Korea University, Seoul, South Korea; ^3^ Department of Internal Medicine, Samsung Medical Center, Sungkyunkwan University School of Medicine, Seoul, South Korea

**Keywords:** multiple myeloma, galectin-9 (Gal-9), programmed death-ligand 1 (PD-L1), plasma cells, prognosis

## Abstract

Galectin-9 (Gal-9) expression can be negatively or positively associated with cancer patient prognosis, depending on the cancer type. However, the nature of this relationship remains unclear in multiple myeloma. Therefore, we evaluated the prognostic value of Gal-9 and its relationship with the expression of PD-L1 molecule, the most widely studied immune checkpoint inhibitor, in patients with newly diagnosed multiple myeloma. Gal-9 and PD-L1 levels in bone marrow aspirate samples were evaluated using immunofluorescence assays. Gal-9 positivity was defined as having ≥1% Gal-9-expressing plasma cells. PD-L1 expression was categorized as low or high based on its median value. The median OS of patients with positive and negative Gal-9 expression was 42 months and not reached, respectively. However, no significant difference was observed in OS between the two groups (*P =* 0.10). Patients with high PD-L1 expression had OS times of 14 and 43 months in the positive and negative Gal-9 expression groups, respectively. In the high PD-L1 expression group, patients expressing Gal-9 had significantly worse OS than those negative for it (*P* = 0.019). Multivariable Cox analysis confirmed that Gal-9 expression could independently predict shortened OS (hazard ratio, 1.090; 95% confidence interval, 1.015–1.171; *P* = 0.018) in patients with high PD-L1 expression. However, in the low PD-L1 expression group, patients with high Gal-9 expression exhibited a trend toward better OS (*P* = 0.816). Our results indicate that the prognostic value of Gal-9 may be related to PD-L1 expression in patients with newly diagnosed multiple myeloma.

## Introduction

Galectin-9 (Gal-9) is differentially expressed in tissues involved in the immune system (1). Gal-9 is involved in diverse biological processes, including the regulation of cell adhesion, migration, polarity, chemotaxis, proliferation, apoptosis, and differentiation (1, 2). The pleiotropic effects of galectins also play an important role in the bone marrow and microenvironment during hematopoiesis (2). Moreover, several studies have shown that Gal-9 levels differ between normal and tumor tissues, and it has been implicated in several aspects of cancer progression (3–5). Previous studies have suggested an inverse correlation between Gal-9 expression and cancer progression in several solid tumors, including breast, gallbladder, and colon cancers, as well as cervical squamous cell carcinoma and hepatocellular carcinoma (3, 5–8). However, it may also function as a tumor suppressor, as loss of Gal-9 expression is closely associated with increased metastasis and high recurrence in breast, cervical, colon, and gastric cancers (3, 6, 9, 10). Some studies have suggested that the most dominant Gal-9 isoform might prevent metastasis by maintaining tissue integrity and hampering tumor cell migration and extravasation, whereas other Gal-9 isoforms facilitate metastasis (1). Recently, Gal-9 has also been implicated in tumor immune evasion (11) by inducing apoptosis of Tim-3-positive T cells (12, 13), and regulating T cell activity *via* death receptor 3 signaling, which is known to promote immune evasion (14). Considering these heterogeneous impacts of Gal-9 on cancer immunity, the effects of its expression on cancer progression remain unclear.

There has been increasing interest in the prognostic implications of Gal-9 expression in patients with cancer. High Gal-9 expression has been correlated with increased survival of various solid cancers (7–9, 15, 16). For example, in a study analyzing 128 patients with colon cancer, Gal-9 expression was a significant predictor for favorable overall survival (OS) in multivariable analysis (9). In another study on 84 patients with breast cancer, patients with Gal-9-positive tumors showed favorable metastasis-free survival (15). A previous meta-analysis of 2,208 patients with solid cancers showed that high Gal-9 expression was significantly associated with better OS (7). However, several studies have reported inconclusive or even opposing results, possibly due to the heterogeneity of tumors with various origins, the divergent roles of Gal-9 in tumor immunity, the diverse expression profiles of receptors, and/or variability in study designs and sample sizes (7). For instance, high expression of Gal-9 was associated with poor survival and early recurrence in a study of renal cell carcinoma involving 196 patients (17). Similarly, a small retrospective study on 48 patients with small cell lung cancer showed that an elevated Gal-9 level was an independent indicator of decreased OS (18). Gal-9 expression was significantly higher in patients with acute myeloid leukemia who experienced treatment failure compared to those in complete remission (19). However, other studies have reported no correlation between Gal-9 expression and cancer prognosis (7, 8). The prognostic roles of galectin family members have also been studied in multiple myeloma. A previous study reported no increase in the serum *LGALS1* level in multiple myeloma and no association with its prognosis (20). However, another study reported that *LGALS1* expression in multiple myeloma cells was upregulated under hypoxic conditions and that downregulation significantly reduced the tumor burden in a mouse model (21). Hence, further studies are required to determine the prognostic role of *LGALS1* in multiple myeloma. Moreover, a study analyzed *LGALS8* expression in patients with multiple myeloma and found that OS was significantly longer in patients with low *LGALS8* levels than in those with high levels (22). Thus, *LGALS8* may be an adverse prognostic predictor for multiple myeloma; however, the prognostic implications of its expression remain unclear.

PD-L1 molecule (PD-L1) plays an important role in mediating immune responses and tumor tolerance by binding to programmed cell death 1 (PD-1) on T lymphocytes and subsequently promoting T cell exhaustion, apoptosis, and the selective suppression of tumor-specific T cells (23, 24). Indeed, PD-L1 expression is a promising prognostic biomarker for several cancers (25, 26) that can predict treatment resistance and unfavorable prognosis. However, it remains unclear whether this relationship exists in multiple myeloma (27). The PD-1/PD-L1 and Tim-3/Gal-9 axes are two major pathways in this area, and their contributions have been documented in various malignancies. Several studies have reported that Gal-9 and PD-L1 serve as independent prognostic biomarkers in solid tumors (16, 28, 29). Meanwhile, a study on 154 patients with hepatocellular carcinoma found that Gal-9 and PD-L1 were expressed in 82.9 and 78.8% of tumor cells, respectively. Multivariable analysis has also indicated that low levels of Gal-9 or PD-L1 are significantly associated with poor survival, with both levels being independent predictors of poor survival (16). Additionally, low levels of circulating Gal-9 or PD-L1 were significantly associated with poor survival in 81 patients with hepatocellular carcinoma analyzed *via* enzyme-linked immunosorbent assay. However, multivariable analysis identified circulating Gal-9 as a significant independent predictor of survival, but not PD-L1 (29). In another study on patients with lung cancer, tumors expressing high levels of Gal-9 or low levels of PD-L1 trended toward poor survival, although no significant differences were observed (28). However, the prognostic significance of Gal-9 in relation to PD-L1 has not been reported for multiple myeloma.

Therefore, we evaluated whether Gal-9 is associated with the survival and prognosis of patients with multiple myeloma. We also analyzed whether Gal-9 expression is affected by the expression of PD-L1. The prognostic value of Gal-9 was evaluated as a potential immune biomarker in association with PD-L1 in patients with newly diagnosed multiple myeloma.

## Materials and Methods

### Patient Cohorts

We evaluated patients with newly diagnosed multiple myeloma who underwent bone marrow aspiration at the Korea University Anam Hospital. The retrospective cohort involved 119 patients with multiple myeloma admitted from January 2011 to April 2019. Of these, 10 patients were excluded because bone marrow samples were unavailable, and 109 bone marrow specimens obtained at the time of diagnosis were examined. All patients included in this study were diagnosed with multiple myeloma according to the International Myeloma Working Group criteria (30). The study protocol was approved by the institutional review board of the Korea University Medical Center. The patients provided written informed consent to participate in this study.

### Immunofluorescence Staining

Tissue sections of 4–5 µm thickness were prepared from formalin-fixed and paraffin-embedded specimens. The sections were then deparaffinized and rehydrated. Prior to staining, they were boiled in sodium citrate buffer (pH 6.0) in a pressure cooker for 10 min. Subsequently, the sections were permeabilized using 0.5% Triton X-100 and blocked by incubating in 5% normal donkey serum for 1 h at room temperature. They were then incubated overnight at 4°C with the following primary antibodies: anti-CD138 (1:100; R&D Systems, Minneapolis, MN, USA), anti-Gal-9 (D9R4A, 1:200; Cell Signaling Technology, Danvers, MA, USA), and anti-PD-L1 (ABM4E54, 1:100; Abcam, Cambridge, UK). The targets were detected using the following fluorochrome-conjugated secondary antibodies (1:200; Invitrogen, Carlsbad, CA, USA) for 1 h at room temperature: Alexa Fluor 488 for CD138, Alexa Fluor 555 for Gal-9, and Alexa Fluor 647 for PD-L1. Isotype-matched antibodies were used as negative controls, and a DAPI-containing mounting medium (ProLong Diamond Antifade Mountant with DAPI; Invitrogen) was used to stain the nuclei.

### Quantitative Real-Time PCR (qPCR)

Total RNA was extracted from bone marrow plasma cells using LaboPass Labozol reagent (Cosmo Genetech, Seoul, Korea). Complementary DNA (cDNA) was reverse transcribed from 1 μg of total RNA using LaboPass cDNA Synthesis kit (Cosmo Genetech) according to the manufacturer’s instructions. qPCR was performed using Labopass SYBR Green Q Master (Cosmo Genetech) in a CFX96 Touch Real-Time PCR Detection System (Bio-Rad, Hercules, CA, USA) according to the manufacturer’s instructions. Glyceraldehyde 3-phosphate dehydrogenase (*GAPDH*) was used as an internal control. Primers used were as follows: *LGALS9*, 5′-AGC TTC TCA GTG TGG ATC TTG-3′ and 5′-TCC TCA GGC GAT GGT AGT AT-3′. Each cDNA sample was tested in triplicate. The cycling conditions included initial template denaturation at 95°C for 3 min, followed by amplification for 40 cycles of 95°C for 10 s, 62°C for 20 s (for *LGALS9*) or 58°C for 10 s (for *GAPDH*), and 72°C (for *LGALS9*) or 68°C (for *GAPDH*) for 20 s. A final melting curve analysis was performed from 65 to 95°C at a rate of 0.05°C/s. The relative *LGALS9* transcript level was determined using the 2^−ΔCt^ method.

### Western Blotting Analysis

Bone marrow samples were lysed with radioimmunoprecipitation assay lysis buffer and centrifuged at 3,000 rpm for 10 min. Loading buffer was added to the cell lysates, which were boiled for 5 min at 95°C. Total protein (20 μg) was resolved through sodium dodecyl sulfate polyacrylamide gel electrophoresis and then transferred onto polyvinylidene fluoride membranes (Bio-Rad, Hercules, CA, USA). Membranes were blocked in 5% bovine serum albumin and incubated with primary anti-Gal-9 (D9R4A, 1:500; Cell Signaling Technology, Danvers, MA, USA) and anti-PD-L1 (ABM4E54, 1:1,000; Abcam, Cambridge, UK) antibodies overnight at 4°C in Tris-buffered saline with 0.1% Tween 20. After washing, membranes were incubated in horseradish peroxidase-conjugated antibodies (1:1,000 for Gal-9 and 1:3,000 for PD-L1, Sigma-Aldrich, Munich, Germany) for 1 h at room temperature. Bands were visualized using Amersham ECL prime Western Blotting Detection Reagents (GE Healthcare, UK). Quantifications of western blots were performed in ImageJ (US National Institutes of Health, Bethesda, MD, USA), with densitometric values normalized to GAPDH and beta-actin.

### Immunofluorescence Analysis

**Figure 1:**
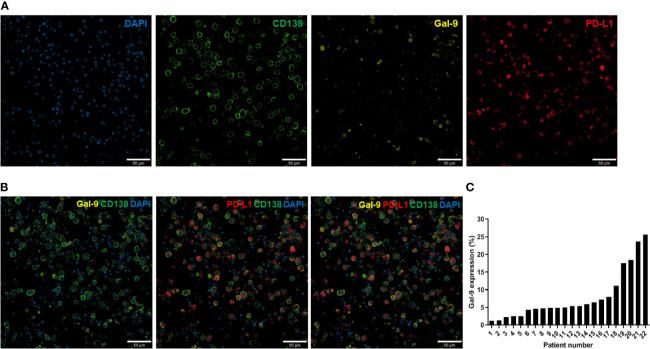
Immunofluorescence analysis of Gal-9 and PD-L1 expression in bone marrow plasma cells from patients with multiple myeloma. **(A)** Confocal images of 4–5-µm-thick formalin-fixed, paraffin-embedded bone marrow aspirate specimens (clot sections) obtained from patients with myeloma. The sections were stained for CD138, Gal-9, and PD-L1. Original magnification, 200×. **(B)** Representative merged images of Gal-9, PD-L1, and CD138 staining are shown. **(C)** Distribution of Gal-9 expression in patients with multiple myeloma.

Immunofluorescence analysis was performed as previously described (31). For all samples, at least two fields (median: 5) per slide were analyzed at 200× magnification using a confocal laser scanning microscope (LSM 800; Carl Zeiss Microscopy GmbH, Oberkochen, Germany; **Figure 1A**). Images were obtained from the individual fluorescence channels under identical conditions, including the bit depth (8 bit), pinhole size (1 AU), laser power, and exposure. The CD138 signal was used to identify plasma cells. Gal-9 and PD-L1 expression levels were calculated based on their mean fluorescent intensity (MFI) within each plasma cell compartment. All analyses were conducted using Celleste Image Analysis Software (Invitrogen). After subtracting the background intensity, the sum of the MFIs in all plasma cell compartments was divided by the total number of plasma cells. The MFI was normalized by dividing this value by the MFI obtained from the isotype-matched control. **Figure 1B** shows representative images of Gal-9- and/or PD-L1-expressing plasma cells. We defined cells with a normalized MFI of ≥5 as Gal-9-expressing plasma cells, which were observed in 23 patients. Gal-9 positivity was defined by the presence of ≥1% Gal-9-expressing plasma cells, and 22 patients were Gal-9-positive (**Figure 1C**). PD-L1 expression ≥ the normalized median MFI value of 9.78 was considered high, and that <9.78 was considered low. The distributions of Gal-9 and PD-L1 expression are shown in **Supplementary Figure 1**.

### Statistical Analysis

Categorical variables were evaluated using the chi-squared or Fisher’s exact tests, and continuous variables were evaluated using Student’s *t*-test. The OS was calculated from the time of diagnosis until death due to any cause, and progression-free survival (PFS) was calculated from the start of treatment to disease progression or death. Differences in OS and PFS were evaluated by Kaplan–Meier analysis and log-rank test. Cox proportional hazard models were used to analyze the associations between survival outcomes and various prognostic factors. No major violation of the proportional hazards assumption was observed using the Schoenfeld method. All tests were two-sided, and *P*-values <0.05 were considered significant. Statistical analyses were performed in R (version 4.0.2), SPSS statistics version 25.0 (SPSS Inc., Chicago, IL, USA), and GraphPad Prism (version 9.1.0; GraphPad, Inc., La Jolla, CA, USA).

## Results

### Relationship Between Gal-9 and PD-L1 Expression

**Figure 2:**
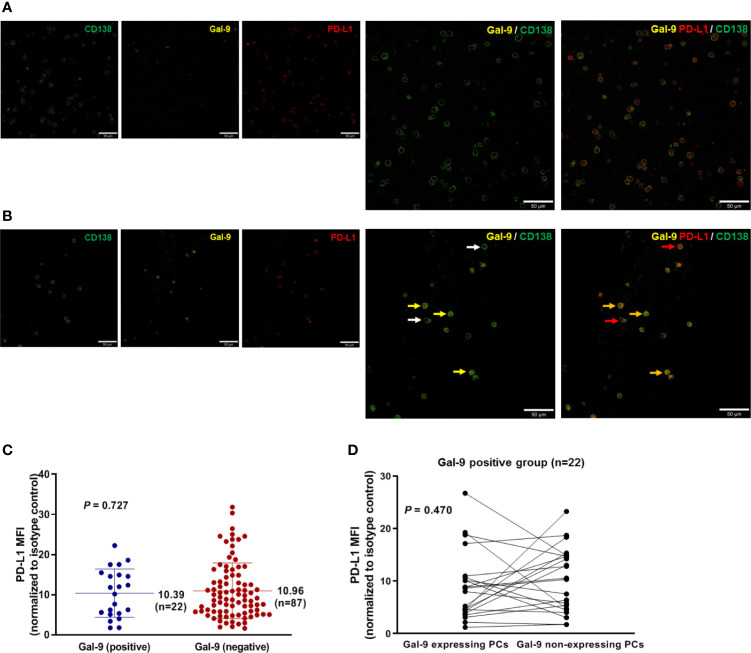
Relationship between Gal-9 and PD-L1 expression. **(A)** Representative Gal-9-negative images are shown. **(B)** Representative Gal-9-positive images are shown. Yellow and white arrows indicate CD138-positive plasma cells with and without Gal-9 expression, respectively. Red arrows indicate PD-L1-expressing CD138-positive plasma cells. Orange arrows indicate CD138-positive plasma cells co-expressing Gal-9 and PD-L1. **(C)** Mean PD-L1 values in the positive and negative Gal-9 expression groups. **(D)** PD-L1 expression in individual cells in patients with positive Gal-9 expression (*n* = 22).

To assess the relationship between Gal-9 and PD-L1 expression, we performed immunofluorescence staining to detect these two proteins in bone marrow samples isolated from newly diagnosed patients with multiple myeloma (**Figures 2A, B**). The mean PD-L1 values in patients with positive (*n* = 22) and negative (*n* = 87) Gal-9 expression were 10.39 (95% CI, 7.73–13.05) and 10.96 (95% CI, 9.48–12.44), respectively (**Figure 2C**), with no significant difference observed between the two groups (*P =* 0.727). In the 22 patients with positive Gal-9 expression, we compared PD-L1 expression between Gal-9 expressing and non-expressing plasma cells. The PD-L1 value of Gal-9-expressing plasma cells (mean, 8.880; 95% CI, 6.00–11.76) was lower than that of Gal-9 non-expressing plasma cells (mean, 10.27; 95% CI, 7.55–12.98); however, these results were not significant (**Figure 2D**; *P =* 0.470). Therefore, no significant association was found between Gal-9 and PD-L1 expression.

### Gal-9 and PD-L1 Expression Analysis Using Different Detection Methods

**Figure 3:**
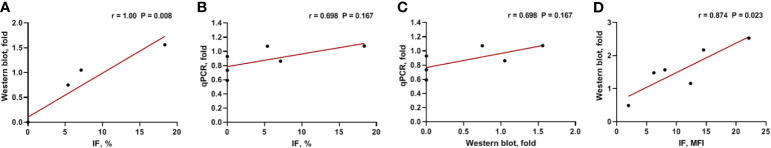
Correlation between Gal-9 and PD-L1 expression results using different detection methods. **(A)** Western blotting *vs.* IF, **(B)** qPCR *vs.* IF, **(C)** qPCR *vs.* western blotting in Gal-9 analysis, and **(D)** Western blotting *vs.* IF in PD-L1 analysis. IF, immunofluorescence; qPCR, quantitative real-time PCR.

We analyzed Gal-9 and PD-L1 expression in the six available bone marrow samples through immunofluorescence, western blotting, and qPCR (**Table 1**). The original western blotting and qPCR data are shown in **Supplementary Figures 2**, **3**, and **Supplementary Table 1**. Immunofluorescence analysis revealed that three patients expressed Gal-9 and three did not; these results were consistent with those of western blotting analysis. We next examined the correlations between the Gal-9 expression results using the different detection methods through Spearman correlation analysis. Gal-9 expression results obtained from western blotting were significantly correlated with those obtained from the immunofluorescence assay (*r* = 1.00; *P* = 0.008; **Figure 3A**). The *LGALS9* gene expression evaluated using qPCR showed a trend of positive correlation with the protein expression results of immunofluorescence (*r* = 0.698; *P* = 0.167; **Figure 3B**) and western blotting (*r* = 0.698; *P* = 0.167; **Figure 3C**) analyses; however, no significant correlations were observed between Gal-9 protein and *LGALS9* gene expression. Then, we examined the correlations between the PD-L1 expression results in the immunofluorescence and western blotting by Pearson correlation analysis. PD-L1 expression results obtained from western blotting were significantly correlated with those obtained from the immunofluorescence (*r* = 0.847; *P* = 0.023; **Figure 3D**).

**Table 1:** Detection of Gal-9 and PD-L1 expression using different methods.

Patient	Gal-9			PD-L1	
	IF(%)	Western blot (fold, Gal-9/GAPDH)	qPCR (fold, *LGALS9/GAPDH*)	IF (MFI)	Western blot (fold, PD-L1/b-actin)
1	0	0	0.733	8.06	1.57
2	0	0	0.928	1.94	0.49
3	18.37	1.56	1.075	6.21	1.48
4	5.38	0.75	1.073	14.55	2.17
5	0	0	0.590	22.15	2.53
6	7.14	1.05	0.862	12.37	1.16

IF, immunofluorescence; MFI, mean fluorescence intensity; qPCR, quantitative real-time PCR.

### Baseline Characteristics in Relation to Gal-9 Expression

Patient characteristics are shown in **Table 2**. No significant difference was observed in median age between patients with positive Gal-9 expression (66; range, 44–77 years) and negative Gal-9 expression (66; range, 43–86 years; *P* = 0.920). In total, 10 patients (45.5%) with positive Gal-9 expression and 38 patients (43.7%) with negative Gal-9 expression had high β2-microglobulin levels (≥5.5 mg/L; *P =* 0.881), whereas 12 patients with positive Gal-9 expression and 42 patients with negative Gal-9 expression showed high PD-L1 expression (≥median; 54.5% vs. 48.3%; *P =* 0.599, respectively). Most patients were in stage II according to the Revised International Staging System classification (*n* = 71; 65.1%) in both the positive (*n* = 11; 50.0%) and negative (*n* = 60; 69.0%) Gal-9 expression groups. Initial combinational treatment with bortezomib, melphalan, and prednisone was administered to 13 patients (59.1%) with Gal-9 expression and 45 patients (51.7%) without Gal-9 expression. Five (22.7%) and 29 (33.3%) patients in the Gal-9-positive and -negative groups, respectively, were treated with autologous stem cell transplantation (*P* = 0.337). The baseline patient characteristics were well-balanced between the positive and negative Gal-9 expression groups, and no significant differences were observed between groups.

**Table 2:** Baseline characteristics and clinical parameters according to galectin 9 expression status.

	Total (*n* = 109)	Negative (*n* = 87)	Positive (*n* = 22)	*P*
Age, years	66 (43–86)	66 (43–86)	66 (44–77)	0.920
≥70	41 (37.6)	33 (37.9)	8 (36.4)	0.892
ECOG performance status				
≥2	6 (5.5)	5 (5.7)	1 (4.5)	1.000
Serum M-protein				
≥3.0 g/dL	51 (46.8)	41 (47.1)	10 (45.5)	0.888
BM plasma cells, %	33.9 (0.7–94.7)	33.8 (0.7–94.7)	37.4 (0.8–90.0)	0.566
Albumin				
<3.5 mg/L	60 (55.0)	50 (57.5)	10 (45.5)	0.311
β2-microglobulin				
≥5.5 mg/L	48 (44.0)	38 (43.7)	10 (45.5)	0.881
LDH				
≥upper normal range	49 (45.0)	37 (42.5)	12 (54.5)	0.311
Cytogenetic abnormalities				
High-risk*	35 (32.1)	30 (34.5)	5 (22.7)	0.291
t(4;14)	20 (18.3)	17 (19.5)	3 (13.6)	
t(14;16)	3 (2.8)	3 (3.4)	0	
del(17/17p) or *TP53* deletion	13 (11.9)	9 (10.3)	4 (18.2)	
Chromosome 1 abnormalities	7 (6.4)	6 (6.9)	1 (4.5)	
PD-L1 expression				
High (>median)	54 (49.5)	42 (48.3)	12 (54.5)	0.599
Calcium				
>11 mg/dL	8 (7.3)	6 (6.9)	2 (9.1)	1.000
Creatinine				
>2 mg/dL	23 (21.1)	16 (18.4)	7 (31.8)	0.240
Hb				
<10 g/dL	69 (63.3)	52 (59.8)	17 (77.3)	0.128
Bone lesions				
≥1 lesion	93 (85.3)	76 (87.4)	18 (81.8)	0.503
ISS				
Stage I	17 (15.6)	12 (13.8)	5 (22.7)	0.494
Stage II	44 (40.4)	37 (42.5)	7 (31.8)	
Stage III	48 (44.0)	38 (43.7)	10 (45.5)	
R-ISS				
Stage I	10 (9.2)	7 (8.0)	3 (13.6)	0.881
Stage II	71 (65.1)	60 (69.0)	11 (50.0)	
Stage III	28 (25.7)	20 (23.0)	8 (36.4)	
mSMART 3.0				
Standard	61 (56.0)	49 (56.3)	12 (54.5)	0.680
High	48 (44.0)	38 (43.7)	10 (45.5)	
Treatment regimen				
VMP	58 (53.2)	45 (51.7)	13 (59.1)	0.714
VTD	21 (19.3)	18 (20.7)	3 (13.6)	
TD or RD	27 (24.8)	22 (25.3)	5 (22.7)	
Supportive only	3 (2.8)	2 (2.3)	1 (4.5)	
Transplantation				
Auto-SCT	34 (31.2)	29 (33.3)	5 (22.7)	0.337
Allo-SCT	0	0	0	
None	75 (68.8)	58 (66.7)	17 (77.3)	

Data are shown as number (percentage) or median (range).

BM, bone marrow; ECOG, Eastern Cooperative Oncology Group; ISS, International Staging System; Hb, hemoglobin; LDH, lactate dehydrogenase; mSMART, Mayo Stratification of Myeloma and Risk-Adapted Therapy; PD-L1, PD-L1 molecule; RD, lenalidomide/dexamethasone; R-ISS, Revised International Staging System; SCT, stem cell transplantation; TD, thalidomide/dexamethasone VMP, bortezomib/melphalan/prednisone; VTD, bortezomib/thalidomide/prednisone.

*High-risk cytogenetics were defined as t(4;14), t(14;16), del(17/17p), TP53 deletion, or chromosome 1 abnormalities including gain(1q) and del(1p).

### Correlation Between Clinical Parameters and Gal-9 Expression

There was no significant association between Gal-9 expression and the presence of osteolytic lesions (HR, 0.651; 95% CI, 0.186–2.283; *P* = 0.503). To examine cytogenetic abnormalities, we assessed the association between Gal-9 expression with chromosome 17 abnormalities and trisomies (hyperdiploidy). We found a significant negative association (HR, 0.150; 95% CI, 0.039–0.583; *P* = 0.006) between Gal-9 expression and trisomies. Chromosome 17 abnormalities were positively associated with Gal-9 expression with borderline significance (HR, 3.204; 95% CI, 0.847–12.120; *P* = 0.086).

### Survival Analysis in Relation to Gal-9 and PD-L1 Expression Status

**Figure 4:**
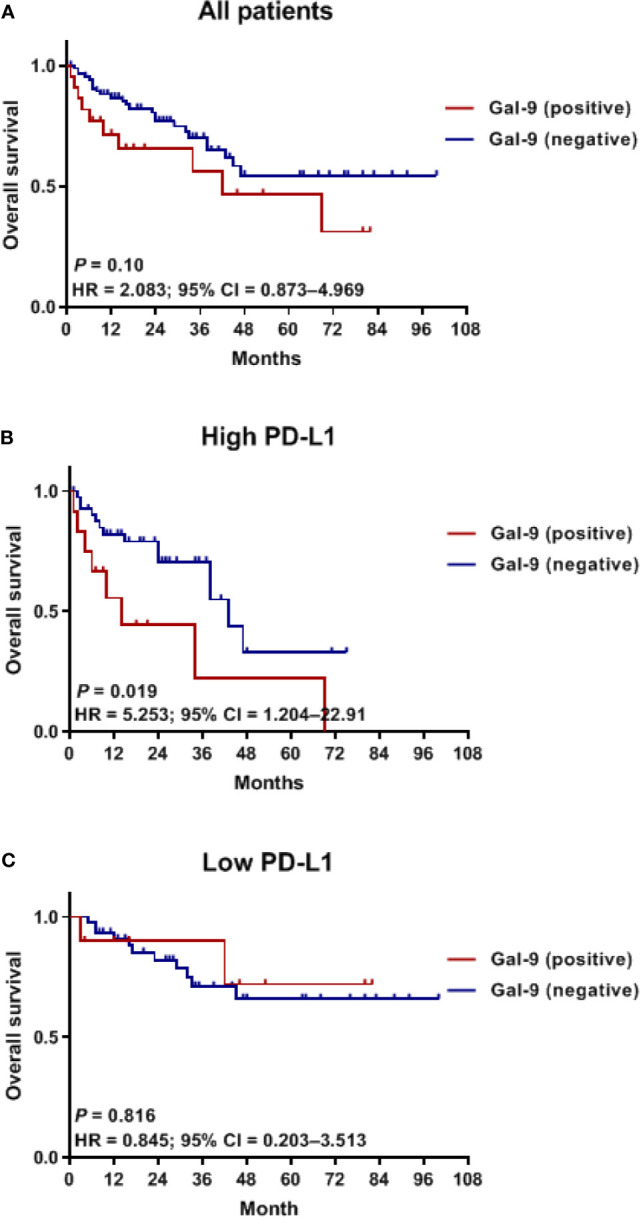
Kaplan–Meier curves for OS in relation to Gal-9 and PD-L1 expression. OS curves in **(A)** all patients, **(B)** the high PD-L1 expression group, and **(C)** the low PD-L1 expression group. OS, overall survival.

**Figure 5:**
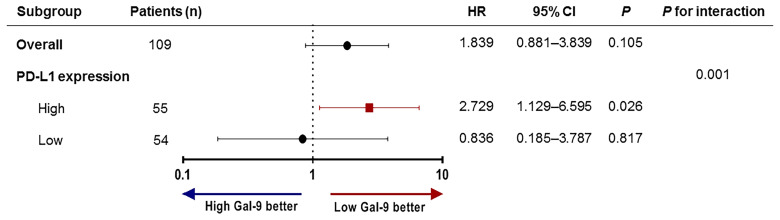
Subgroup analysis and forest plot of OS. Hazard ratios and *P*-values for interactions are based on Cox regression analysis. OS, overall survival.

To assess whether Gal-9 expression was correlated with the survival of patients with multiple myeloma, we evaluated patient survival rates in relation to Gal-9 expression by Kaplan–Meier analysis (**Figure 4A**). The median OS for patients with positive Gal-9 expression was 42 months, while the median OS for those with negative expression was not reached. However, no significant difference in OS was observed between the two groups (*P =* 0.10). When only patients with high PD-L1 expression were analyzed, the OS durations were 14 and 43 months in the positive and negative Gal-9 expression groups, respectively (**Figure 4B**). In the high PD-L1 expression group (*n* = 54), patients with positive Gal-9 expression had significantly longer OS than those with negative Gal-9 expression (*P =* 0.019). However, in the low PD-L1 expression group (*n* = 55), patients with positive Gal-9 expression exhibited a trend of longer OS (*P =* 0.816; **Figure 4C**). Subsequently, we conducted subgroup analysis to evaluate whether the effect of Gal-9 expression differed based on PD-L1 expression (**Figure 5**). The results showed that the unfavorable effect of Gal-9 on OS was significant in patients with high PD-L1 expression (HR, 2.729; 95% CI, 1.129–6.595; *P* = 0.026) but not in those with low PD-L1 expression (HR, 0.836; 95% CI, 0.185–3.787; *P* = 0.817; *P* for interaction = 0.001).

To evaluate the prognostic value of Gal-9 expression for OS in patients with high PD-L1 expression, Cox regression analysis was performed (**Table 3**). Univariable analysis revealed that poor prognosis was significantly associated with lactate dehydrogenase levels ≥ the upper normal limit (*P =* 0.011), and high-risk cytogenetics (*P =* 0.002) which were defined as at least one of the following factors: t(4;14), t(14;16), del(17/17p), tumor protein p53 (*TP53*) deletion, or chromosome 1 abnormalities, including gain(1q) and del(1p). Additionally, poor OS was correlated with old age (≥70 years) and high Gal-9 expression, although not significantly. However, in a multivariable Cox analysis using a backward stepwise elimination method and including all the variables used in the univariable analysis, high Gal-9 expression independently predicted poor OS (HR, 1.090; 95% CI, 1.015–1.171; *P* = 0.018) in patients with high PD-L1 expression.

**Table 3:** Univariable and multivariable analyses of OS in the high PD-L1 expression group.

Prognostic factors	Univariable				Multivariable		
	HR	95% CI	*P*		HR	95% CI	*P*
Age (≥70)	2.261	0.859, 5.950	0.098		5.042	1.636, 15.543	0.005
ECOG performance status (≥2)	0.754	0.100, 5.701	0.785				
Serum M-protein (≥3 mg/dL)	1.206	0.514, 2.828	0.667				
Serum FLC ratio (≥100)	0.876	0.350, 2.191	0.777				
BM plasma cell, %	1.004	0.991, 1.017	0.525				
β2-microglobulin (≥5.5 mg/L)	1.819	0.732, 4.521	0.198				
Albumin (<3.5 g/dL)	1.101	0.467, 2.591	0.826				
LDH (≥upper normal range)	3.170	1.301, 7.724	0.011		3.572	1.389, 9.187	0.008
Cytogenetics (high-risk*)	4.226	1.662, 10.743	0.002		5.180	1.944, 13.802	0.001
Gal-9 expression	1.049	0.981, 1.123	0.163		1.090	1.015, 1.171	0.018

BM, bone marrow; ECOG, Eastern Cooperative Oncology Group; FLC, free light chain; LDH, lactate dehydrogenase.

*High-risk cytogenetics were defined as t(4;14), t(14;16), del(17/17p), TP53 deletion, or chromosome 1 abnormalities including gain(1q) and del(1p).

## Discussion

This is the first study to indicate that the prognostic value of Gal-9 depends upon PD-L1 expression in patients with multiple myeloma. Positive Gal-9 expression in bone marrow plasma cells was associated with poor OS in patients with high PD-L1 expression, and negative Gal-9 expression showed a trend toward better OS in the high PD-L1 expression group. Multivariable analysis confirmed that positive Gal-9 expression independently predicted worse OS in patients with high PD-L1 expression.

Several recent studies evaluated the prognostic value of Gal-9 in patients with solid tumors (6, 10). However, these studies showed contradictory results. Some studies showed that high Gal-9 expression was related to better survival in patients with solid cancer, including colon cancer, gastric cancer, and hepatocellular carcinoma, whereas other studies reported no association (7). Moreover, the precise mechanism by which Gal-9 presumably improves the survival of patients with cancer remains unknown. Its role in enhancing cell aggregation may be one of the underlying mechanisms. We examined Gal-9 expression in four samples of extramedullary plasmacytoma in the skull, neck node, chest wall, and scalp; none expressed Gal-9 (**Supplementary Figure 4**). Although a small number of samples was analyzed, this lack of Gal-9 expression may be associated with extramedullary disease. Evaluation of the correlation between Gal-9 and neural cell adhesion molecule 1 expression may help identify the role of Gal-9 in cell aggregation. However, in other aspects of multiple myeloma, interactions between plasma cells and the extracellular matrix can be essential for progression, and plasma cell clumps with enhanced cell-to-cell adhesion may be associated with poor prognosis (32). Another possible mechanism promoting the survival of patients with cancer may involve the role of Gal-9 in regulating apoptosis (1). Recombinant Gal-9 can modulate apoptosis in various malignant cells (7). However, recombinant Gal-9 differs from endogenous Gal-9. The antitumor immune response role of Gal-9 may also influence the survival of patients with cancer (7). Gal-9 expression is known to reinforce the antitumor immunity of T cells, which cooperate with dendritic cells through the Gal-9/Tim-3 pathway (33). This antitumor immune effect of Gal-9 has been observed in a lung cancer mouse model (34). However, high Gal-9 expression has also been closely correlated with tumor immune escape. Several studies reported that Tim-3 promotes CD8+ T cell exhaustion, thus negatively regulating T cell responses and consequently inducing the expansion of myeloid-derived suppressor cells through mechanisms involved in tumor growth and immune evasion (35). Therefore, how Gal-9 expression contributes to immune evasion and antitumor activity in patients with cancer remains unclear. Additionally, whether Gal-9 enhances or suppresses cancer progression is controversial, and further studies are needed to clarify these issues.

High Gal-9 expression reportedly predicts both favorable and unfavorable prognoses, depending on the cancer type (7). An experimental study reported that recombinant Gal-9 exerts an anti-myeloma effect by inducing the death of primary human multiple myeloma cells (36). However, the prognostic potential of Gal-9 for multiple myeloma OS or PFS remains controversial and should be evaluated in a large-cohort study. We found no significant difference in OS in relation to Gal-9 expression. However, positive Gal-9 expression was significantly associated with poor OS in the high PD-L1 expression group, whereas patients with positive Gal-9 expression exhibited a trend toward improved OS in the low PD-L1 expression group. The prognostic values of Gal-9 and PD-L1 have been studied in solid cancers. Low Gal-9 or PD-L1 levels are associated with poor survival in hepatocellular carcinoma (16, 29). High Gal-9 or low PD-L1 levels tend to be associated with poor survival in lung adenocarcinoma (28). In this study, we found that high Gal-9 or PD-L1 levels are associated with poor survival in multiple myeloma and that Gal-9 appears to have prognostic implications in relation to PD-L1. These findings suggest that PD-L1 expression modulates the effect of Gal-9 on multiple myeloma. For instance, when PD-L1 is highly expressed, the immune evasion role of Gal-9 may predominate. However, with low PD-L1 expression, its antiproliferative or proapoptotic effects may be increased.

PD-L1 also plays an important role in mediating the immune response and tumor tolerance by binding to PD-1 on T lymphocytes and promoting T cell exhaustion, apoptosis, and the selective suppression of tumor-specific T cells (23, 24). Although the interaction between Gal-9 and PD-L1 may affect the role of Gal-9 in relation to PD-L1 expression, we found no evidence that PD-L1 regulated Gal-9 expression in multiple myeloma. Therefore, further studies on the interactions between Gal-9 and PD-L1 or other immune modulators may reveal the prognostic roles of Gal-9 in patients with multiple myeloma.

There are several limitations to this study. First, it was performed using a retrospective cohort and included a relatively small number of patients. Thus, an independent cohort should be evaluated to confirm our results, and long-term and multi-center data are needed to determine whether Gal-9 expression has prognostic implications and whether these are related to PD-L1 expression. Second, no study has reported a threshold value for positive or negative Gal-9 expression during immunofluorescence staining. Although we used 1% or more as a threshold, a more accurate method is required to determine the appropriate value. Third, the most appropriate antibodies and strategies for assessing Gal-9 and PD-L1 levels in bone marrow samples are unclear. We used immunofluorescence staining with the anti-Gal-9 clone D9R4A and anti-PD-L1 clone ABM4E54; however, will be necessary to establish standardized methods. Fourth, we could not perform a deeper molecular analysis. Gene-editing studies including an epigenetic analysis on miRNAs would support the observed link between PD-L1 and Gal-9 on plasma cells. Also, Gal-9 genetic overexpression with functional testing and analysis would help to clarify the correlation between PD-L1 and Gal-9 on plasma cells. Finally, because this study was conducted in patients with newly diagnosed multiple myeloma, our results may not apply to relapsed/refractory multiple myeloma. Further study is needed to evaluate prognostic values of Gal-9 and PD-L1 in relapsed/refractory multiple myeloma.

## Conclusion

Gal-9 expression is associated with poor prognosis in patients with newly diagnosed multiple myeloma that have high PD-L1 expression, indicating that its prognostic value is related to PD-L1 expression. Further studies are required to investigate the prognostic relationships between Gal-9 and other immune checkpoint inhibitors in multiple myeloma.

## Data Availability Statement

The raw data supporting the conclusions of this article will be made available by the authors, without undue reservation.

## Ethics Statement

The studies involving human participants were reviewed and approved by the Korea University Medical Center. The patients/participants provided their written informed consent to participate in this study.

## Author Contributions

B-HL, YP, and BSK designed the study. J-HK, K-WK, and SJK contributed to patient data collection and sample preparation. B-HL, J-HK, and S-JL performed the experiments and analyzed the data. B-HL and BSK contributed to the interpretation of the data. B-HL wrote the manuscript. BSK revised the manuscript. All authors contributed to the article and approved the submitted version.

## Funding

This research was supported by the Bio and Medical Technology Development Program of the National Research Foundation, funded by the Ministry of Science and ICT (NRF-2017M3A9C8060403) and a grant from the Korea Health Technology R&D Project through the Korea Health Industry Development Institute, funded by the Ministry of Health & Welfare, Republic of Korea (grant number: HI17C2072). The funders had no role in study design, data collection and analysis, decision to publish, or preparation of the manuscript.

## Conflict of Interest

The authors declare that the research was conducted in the absence of any commercial or financial relationships that could be construed as a potential conflict of interest.
