# Supplementary material for: Prognostic Value of Galectin-9 Relates to Programmed Death-Ligand 1 in Patients With Multiple Myeloma
Source: Front Oncol. 2021 Jun 14;11:669817. doi: 10.3389/fonc.2021.669817 (PMC8238373; doi:10.3389/fonc.2021.669817)
Supplement: Supplementary file 1 [file DataSheet_1.docx]

Supplementary Material

# Supplementary Table

**Supplementary Table 1.** Gal-9 gene expression by qPCR in multiple myeloma patients

# Supplementary Figure


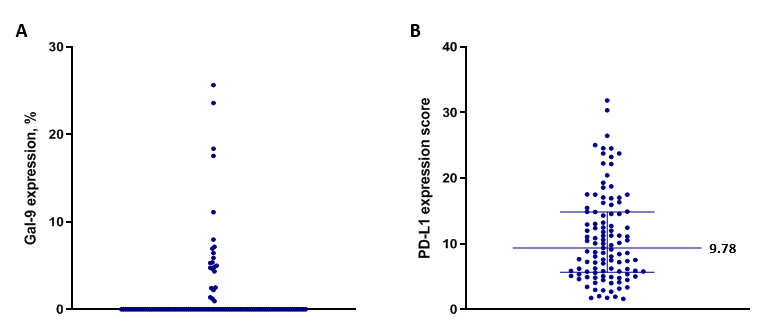


**Supplementary Figure 1.** The distribution of the (A) Gal-9 and (B) PD-L1 expression in patients with multiple myeloma (n =109).


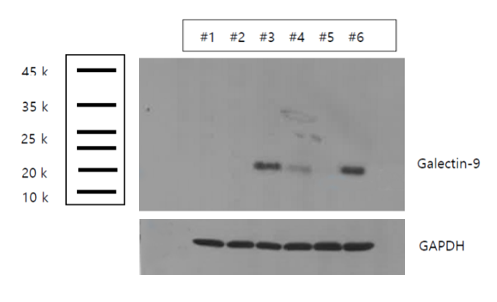


**Supplementary Figure 2.** Western blot analysis of Gal-9 expression. Among 6 patients, 3 patients showed Gal-9 expression.


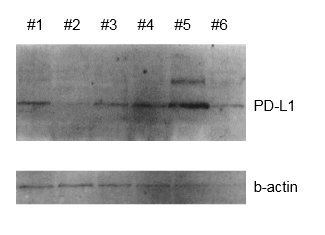


**Supplementary Figure 3.** Western blot analysis of PD-L1 expression.


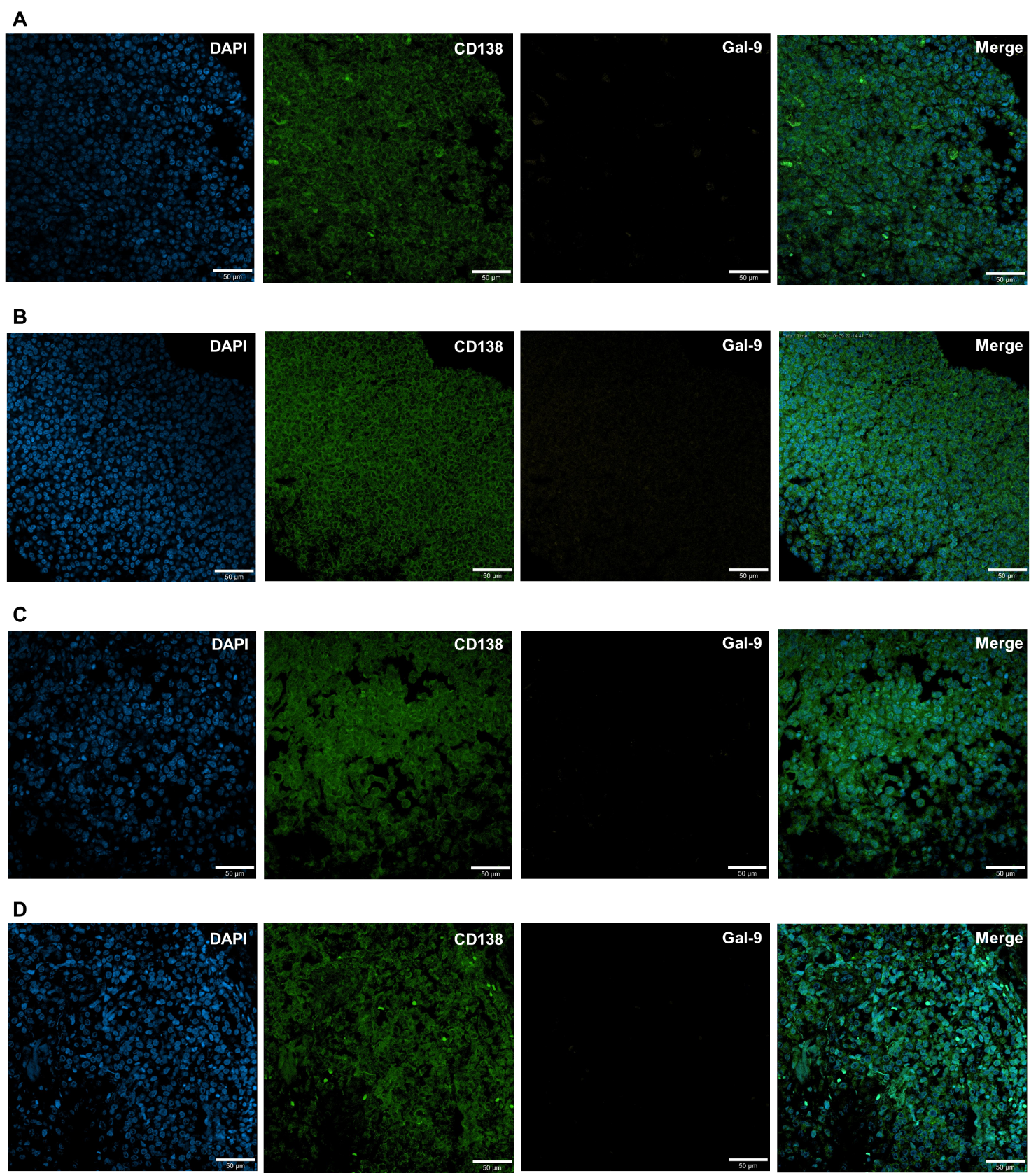


**Supplementary Figure 4.** Immunofluorescence analysis of Gal-9 expression in extramedullary plasmacytoma from patients with multiple myeloma. Formalin-fixed, paraffin-embedded specimens were sectioned at 4–5 µm. The sections were then incubated with antibodies to CD138 (1:100) and Gal-9 (1:200) followed by incubation with the appropriate secondary antibodies (Alexa Fluor 488, 1:200 and Alexa Fluor 555, 1:200) at room temperature for one hour. Nuclei were counterstained using DAPI, and all images were captured using a confocal laser scanning microscope (CLSM 800, Carl Zeiss Microscopy GmbH). Original magnification ×200. Representative images examined in (A) skull, (B) neck node, (C) chest wall, and (D) scalp.
